# Clinical Features and Gene Expression Patterns in Adults Hospitalized With Respiratory Syncytial Virus and Human Metapneumovirus Infection

**DOI:** 10.1093/infdis/jiaf084

**Published:** 2025-07-16

**Authors:** Colin Samoriski, Chin-Yi Chu, Ann R Falsey, Derick Peterson, Soumyaroop Bhattacharya, Daniel P Croft, Angela R Branche, Michael Peasley, Andrea Baran, Anthony Corbett, John Ashton, Thomas J Mariani, Edward E Walsh

**Affiliations:** Infectious Disease Division, Department of Medicine, University of Rochester, Rochester, New York, USA; Department of Pediatrics and Center for Children's Health Research, University of Rochester, Rochester, New York, USA; Infectious Disease Division, Department of Medicine, University of Rochester, Rochester, New York, USA; Department of Biostatistics and Computational Biology, University of Rochester, Rochester, New York, USA; Department of Pediatrics and Center for Children's Health Research, University of Rochester, Rochester, New York, USA; Pulmonary and Critical Care Medicine Division, Department of Medicine, University of Rochester, Rochester, New York, USA; Infectious Disease Division, Department of Medicine, University of Rochester, Rochester, New York, USA; Infectious Disease Division, Department of Medicine, University of Rochester, Rochester, New York, USA; Department of Biostatistics and Computational Biology, University of Rochester, Rochester, New York, USA; Department of Biostatistics and Computational Biology, University of Rochester, Rochester, New York, USA; Genomics Research Center, University of Rochester, Rochester, New York, USA; Department of Pediatrics and Center for Children's Health Research, University of Rochester, Rochester, New York, USA; Infectious Disease Division, Department of Medicine, University of Rochester, Rochester, New York, USA

**Keywords:** RSV, HMPV, adults, gene expression, clinical features

## Abstract

**Background:**

Respiratory syncytial virus (RSV) and human metapneumovirus (HMPV) cause acute respiratory infections (ARI) in persons of all ages. The viruses are structurally similar although HMPV lacks 2 nonstructural proteins of RSV known to suppress interferon signaling.

**Methods:**

We analyzed data from 2 studies that prospectively enrolled hospitalized adults with ARI and compared clinical characteristics and outcomes of HMPV and RSV infection. Gene expression was compared between subjects with HMPV and RSV using DESeq2 to analyze read counts from samples of globin-reduced RNA from whole blood, sequenced using Illumina NovaSeq.

**Results:**

Of 1914 illnesses evaluated, 127 (6.6%) had RSV and 90 (4.7%) had HMPV identified as a sole viral pathogen. Demographics and preexisting conditions were similar although HMPV patients had significantly less active tobacco use and numerically less underlying heart and lung disease. Sore throat, temperature >38°C, and clinically adjudicated pneumonia were significantly more frequent with HMPV compared to RSV. Restricting analysis to those subjects adjudicated as viral alone without bacterial coinfection, we identified 197 differentially expressed genes between RSV- and HMPV-infected subjects. Genes with higher expression in HMPV-infected individuals were associated with antigen binding, immunoglobulin production, and adaptive immunity. Genes increased in RSV-infected individuals were associated with NK T cells.

**Conclusions:**

Although RSV and HMPV are closely related viruses, we found differences in the clinical features of adults with HMPV patients presenting with more flu-like symptoms and viral pneumonia. Peripheral blood gene expression of hospitalized HMPV-infected patients also differed compared to those hospitalized and infected with RSV.

Respiratory syncytial virus (RSV) and human metapneumovirus (HMPV) are closely related, enveloped, negative-sense, single-stranded RNA viruses in the Pneumoviridae family that cause acute respiratory infections (ARI) in children and adults throughout life [1–3]. The viruses are genetically and structurally similar, with the fusion (F) protein of HMPV and RSV sharing approximately 36% sequence homology. Each F protein has at least 6 primary antigenic sites (∅ to V) with some cross reactivity between site III of each virus [4, 5]. A distinguishing feature between the viruses is that HMPV lacks 2 nonstructural proteins expressed by RSV that are potent suppressors of type I interferon activity [6].

In recent years, the burden of RSV in adults has become more well defined, accounting for 60 000–180 000 hospitalizations and 10 000–14 000 deaths annually in the United States [7, 8]. Less is known about the epidemiology and impact of HMPV in adults but studies to date suggest it is a significant cause of morbidity and mortality [3, 9–12]. Three RSV vaccines for older adults are now licensed, and HMPV vaccines are in development [13–15]. Given the similar winter-time seasonality (in Northern and Southern hemispheres) and populations affected, combination RSV-HMPV vaccines are under consideration. Better understanding of the epidemiology and clinical characteristics of HMPV infection compared to RSV could inform development and implementation of new vaccines. Additionally, distinguishing host response to each virus may have implications for vaccine and therapeutic development.

Therefore, we collected clinical and gene transcriptomic data in hospitalized adults with diagnoses compatible with ARI or acute cardiopulmonary illness during 2 previous National Institutes of Health-funded studies done to develop improved diagnostics for respiratory infections [16, 17]. Clinical features and gene expression profiles were compared between hospitalized patients infected with RSV and those infected with HMPV.

## METHODS

### Study Design

Participants were recruited prospectively over 3 consecutive winters from 2008 to 2011 for study 1 and from 2019 to 2023 for study 2 [17, 18]. Similar inclusion/exclusion criteria (see Supplementary Material) were used for both studies and HMPV and RSV infection were documented by reverse transcriptase-polymerase chain reaction (RT-PCR) and/or a 4-fold rise in RSV or HMPV serum immunoglobulin G (IgG) titers. Whole blood for RNA sequencing and gene expression analysis was only collected in study 2.

### Study Period and Sites

Study subjects were enrolled in Rochester, NY. Study 1 was conducted at Rochester General Hospital (RGH) between November 1 and May 30 each year from 2008 to 2011, and Study 2 was conducted at both the University of Rochester Medical Center (URMC) and RGH between March 2019 and April 2023 with year-round recruitment. Study 2 was paused from March-October 2020 due to the coronavirus disease 2019 (COVID-19) pandemic.

### Recruitment

Potential participants with symptoms of acute cardiopulmonary illness (difficulty breathing, cough, wheezing, chest pain) or admission diagnoses compatible with ARI (ie, pneumonia, acute exacerbations of chronic obstructive pulmonary disease or asthma, bronchitis, upper respiratory infection, influenza, or viral syndrome) were screened by reviewing hospital admission logs Monday through Friday. The primary inclusion criteria included age ≥ 18 years and symptoms or diagnosis consistent with ARI. Exclusion criteria included history of immunosuppressive conditions or medications, use of antibiotics more than 24 hours prior to enrollment, and inability to provide informed consent. A limited number of patients with noninfectious cardiopulmonary diagnoses (pulmonary embolism, congestive heart failure, lung cancer, obstructive sleep apnea, etc.) were included as control cases. Participants were enrolled within 24 hours of admission if hospitalized. The studies were approved by URMC and RGH institutional review boards and participants or their legally authorized representatives signed written informed consent prior to study procedures.

### Acute Illness Evaluation

At enrollment, demographic, medical history, clinical, and laboratory information were collected from medical records and patient and family interviews. Standard of care test results were recorded. Study sample collection included a nasal swab for viral and atypical bacterial pathogens by RT-PCR, sputum if available for bacterial Gram stain and culture and RT-PCR for viral pathogens, and blood for serum procalcitonin testing (VIDAS BRAHMS, Bio-Merieux) if not performed as part of standard of care. Blood cultures, sputum Gram stain, and culture were performed according to standard methods. *Streptococcus pneumoniae* and *Legionella* urine antigen (BinaxNow, Abbott) testing was performed at the discretion of treating staff but captured in the database. In study 1, convalescent sera was collected approximately 30 days postinfection. Subjects in study 2 had a Tempus Blood RNA Tube collected within 24 hours of admission, but acute and convalescent serum was not collected.

### Clinical and Microbiological Adjudication

ARI cases were clinically adjudicated by a panel (E. E. W., A. R. F., D. P. C., A. R. B.) of infectious disease and pulmonary medicine specialists and classified into discrete microbiologic categories of viral infection alone, bacterial infection alone, or bacterial-viral coinfection with definite, probable, or no confidence categorization included. Chest radiographs were reviewed along with the official radiologist reading and cases adjudicated as pneumonia or nonpneumonic ARI (see Supplementary Materials). Clinical adjudication is increasingly accepted for outcomes when there is no clear gold standard [19]. Only cases from study 2 judged unanimously as having definitive microbiology were sent for RNA sequencing and only HMPV and RSV PCR-positive cases were included in this analysis.

### Laboratory Methods

#### Real-time PCR Analysis

For study 1, RT-PCR assays for RSV and HMPV, and multiple other viruses were performed in the research laboratory using published methods [3, 20, 21]. The RT-PCR assays were based on amplification of the RSV fusion protein gene and the HMPV nucleocapsid protein gene, each with sensitivity of 1 plaque forming unit. For study 2, RT-PCR for viral and atypical bacterial pathogens was performed by FilmArray Respiratory Panel or FilmArray PN Panel (BioFire Diagnostics) either as standard of care or in the research laboratory.

#### Viral Serologic Analysis

Immunoglobulin G titers in acute and convalescent serum specimens were determined using established solid-phase immunoassays methods for RSV and HMPV [3, 22–24]. Briefly, for RSV the fusion and G proteins of group A and B RSV were used in the solid phase, and for HMPV sucrose-gradient purified HMPV from group A and B HMPV were used in the solid phase. A ≥ 4-fold rise in viral-specific immunoglobulin G level was considered evidence of infection.

#### RNA Sequencing

Whole blood from Tempus Blood RNA Tubes was collected from 159 subjects (43 HMPV, 54 RSV, 62 noninfected controls), centrifuged, and RNA isolated using the Tempus Spin RNA Isolation Kit (Applied BioSystems). Total RNA was processed for globin reduction using GLOBINclear Human Kit. A cDNA library construction was performed using the TruSeq Stranded mRNA library kit (Illumina) with 200 ng of globin-reduced total RNA. cDNA quantities were determined with the Qubit Fluorometer (Life Technologies) and quality assessed using the Agilent Bioanalyzer 2100. Libraries were sequenced (single-end reads) on the Illumina HiSeq2500 to generate 20 million reads/sample. Reads were aligned using the STAR algorithm and expression values summarized using HTSeq.

A total of 7352 genes showing robust expression, and less than 10% variance associated with the cDNA synthesis reaction in which they were generated, were included in analyses. Gene expression patterns for subjects with HMPV infection and RSV infection were compared using the Wald test, following counts per million normalization, in DESeq2 [25]. Significance for differentially expressed genes was defined at a false discovery rate (FDR) adjusted *P* value of <.05. Canonical pathway identification, ontology, and phenotype functional enrichment analysis was performed using ToppGene Suite (https://toppgene.cchmc.org/).

### Statistical Analysis

Comparisons of clinical variables were conducted using *t* test or 2-tailed Fisher exact test as appropriate. Gene expression patterns for subjects with HMPV and RSV infection were compared using the Wald test in DESeq2 [25].

## RESULTS

During study 1, 1534 hospitalizations for ARI met inclusion criteria and in 842 cases the patient consented to participate. During study 2, 2217 hospitalizations met inclusion criteria for ARI and in 1072 cases the patient consented to participate. All illnesses were tested for both HMPV and RSV. The cases from the 2 studies were similar but had some key differences in baseline characteristics (Table 1). Patients enrolled in study 1 were more likely to have preexisting cardiopulmonary disease and diabetes mellitus, as well having been admitted from a home setting. Participants in the second study were significantly younger, more racially diverse, and had a higher incidence of asthma, chronic kidney disease, substance abuse, and dementia.

**Table 1.** Comparison of Baseline Characteristics of the 2 Parent Studies

Characteristic	Study 1 (n = 842)	Study 2 (n = 1072)	*P* Value
Age, y, mean (SD)	64.9 (16.8)	62.2 (16.6)	.0005
Female sex, No. (%)	445 (52.9)	585 (54.6)	.460
Race, No. (%)			
White	635 (75.4)	726 (67.7)	.0003
Black	192 (22.8)	316 (29.4)	.001
Asian	4 (0.48)	3 (0.28)	.710
Other	11 (1.3)	27 (2.5)	.069
Ethnicity, No. (%)			
Hispanic	103 (12.2)	106 (10.0)	.105
Residence, No. (%)			
Home	774 (91.9)	950 (88.6)	.017
Assisted living	42 (5.0)	76 (7.1)	.68
Nursing home	26 (3.1)	16 (1.5)	.70
Other	0	30 (2.8)	<.0001
Active tobacco use, No. (%)	244 (29.0)	345 (32.2)	.135
Chronic medical condition, No. (%)			
COPD	350 (41.6)	431 (40.2)	.574
Asthma	209 (24.8)	382 (35.6)	<.0001
Coronary artery disease	247 (29.3)	211 (19.7)	<.0001
Congestive heart failure	248 (29.5)	164 (15.3)	<.0001
Diabetes mellitus	303 (36.0)	333 (31.1)	.025
Chronic kidney disease	42 (5.0)	88 (8.2)	.006
Substance use	14 (1.7)	120 (11.2)	<.0001
Dementia	7 (0.8)	34 (3.2)	.0004

Abbreviation: COPD, chronic obstructive pulmonary disease.

RSV was detected in 69 (8.2%) and HMPV in 49 (5.8%) cases in study 1 and in 63 (5.9%) and 46 (4.3%) cases in study 2, respectively. Five cases in study 1 had evidence of mixed RSV-HMPV infection and were excluded from further analysis. Combining the 2 studies resulted in a total of 1914 ARI hospitalizations evaluated, 127 (6.6%) with RSV and 90 (4.7%) with HMPV as single viral infections available for the comparative analysis. Ninety percent of participants with HMPV and 87% of those with RSV were diagnosed by RT-PCR with remaining cases diagnosed by serology alone.

### Clinical Characteristics

Mean age, sex, race, and ethnicity of participants infected with RSV compared with HMPV-infected patients were similar, but some clinical variables differed between the 2 infected populations (Table 2). Patients infected with HMPV had significantly less active tobacco use (17.8% vs 30.7%, *P* = .04) and higher body mass index (33.0 vs 29.1, *P* = .02) compared to those infected with RSV. A greater proportion of HMPV-infected participants lived in the community rather than in congregate settings (assisted living or nursing home) with numerically lower rates of underlying chronic obstructive pulmonary disease, congestive heart failure, and substance abuse in HMPV-infected patients than in those with RSV. The prevalence of asthma, diabetes mellitus, chronic kidney disease, and malignancy did not differ between patients with RSV and HMPV.

**Table 2.** Comparison of RSV and HMPV Demographic and Baseline Characteristics

Characteristic	RSV (n = 127)^a^	HMPV (n = 90)^b^	*P* Value
Age, y, mean (SD)	66.8 (17.6)	65.2 (17.8)	.51
Female sex, No. (%)	70 (55.1)	52 (57.8)	.78
BMI, kg/m^2^, mean (SD)	29.1 (8.8)	33.0 (12.2)	.02
Race, No. (%)			
White	99 (78.0)	69 (76.7)	.87
Black	27 (21.3)	19 (21.1)	1.0
Other	1 (0.80)	2 (2.2)	.57
Ethnicity, No. (%)			
Hispanic	15 (11.8)	13 (14.4)	.68
Residence, No. (%)			
Home	104 (81.9)	82 (91.1)	.08
Assisted living	13 (10.2)	6 (6.7)	.47
Nursing home	6 (4.7)	1 (1.1)	.24
Other	4 (3.2)	1 (1.1)	.41
Active tobacco use, No. (%)	39 (30.7)	16 (17.8)	.04
Chronic medical condition, No. (%)			
COPD	52 (40.9)	28 (31.5)	.15
Asthma	40 (31.5)	28 (31.1)	1.0
Coronary artery disease	35 (27.6)	24 (26.7)	1.0
Congestive heart failure	33 (26.0)	16 (17.8)	.18
Diabetes mellitus	44 (34.6)	32 (35.6)	.89
Chronic kidney disease	7 (5.5)	5 (5.6)	1.0
Substance use disorder	9 (7.1)	2 (2.2)	.13
Dementia	4 (3.2)	3 (3.3)	1.0
Any medical condition	126 (99.2)	88 (97.8)	.57
Number of medical conditions, mean (SD)	4.0 (2.1)	3.8 (1.9)	.28

Abbreviations: BMI, body mass index; COPD, chronic obstructive pulmonary disease; HMPV, human metapneumovirus; RSV, respiratory syncytial virus; RT-PCR, reverse transcriptase-polymerase chain reaction.

^a^In study 1 in which serology was available, 25% of RSV infections were seropositive only, 25% were RT-PCR–positive only, and 50% were both RT-PCR and seropositive.

^b^In study 1 in which serology was available, 20% of HMPV infections were seropositive only, 41% were RT-PCR–positive only, and 39% were both RT-PCR and seropositive.

Illness symptoms and clinical outcomes also differed between RSV- and HMPV-infected cases (Table 3). Infection with HMPV was more often associated with sore throat (50.0% vs 33.9%, *P* = .02), subjective feverishness (64.4% vs 48.0%, *P* = .02), and measured temperature >38.0°C (33.3% vs 21.3%, *P* = .05) with numerically more constitutional symptoms and history of rigors compared to RSV-infected participants. RSV-infected patients more often complained of wheezing and shortness of breath with significantly increased rates of confusion (14.2% vs 4.4%, *P* = .02) and supplemental oxygen use (32.3% vs 17.8%, *P* = .02) compared to HMPV-infected patients. RSV-infected patients had numerically higher rates of intensive care unit admission, mechanical ventilation, and longer lengths of hospital stay (mean 6.4 days vs 4.4 days), but these differences were not statistically significant. There were no distinguishing differences in laboratory values or chest radiograph findings between cases with RSV and HMPV. Frequently, multiple findings were noted with vague interpretations (ie, findings that could represent atelectasis, pneumonia, or edema) in both groups. Antibiotic use was similar, with more than 80% of hospitalized patients with RSV and HMPV receiving antibiotics. In-hospital mortality was uncommon but was similar between RSV and HMPV infections at 2.4% versus 2.2%, respectively.

**Table 3.** Signs and Symptoms at Admission, Clinical Characteristics, and Outcomes

	RSV (n = 127)	HMPV (n = 90)	*P* Value
Symptom, No. (%)			
Illness duration preenrollment, d, mean (SD)	6.2 (4.8)	5.9 (4.8)	.68
Nasal congestion	88 (69.3)	64 (71.1)	.88
Sore throat	43 (33.9)	45 (50.0)	.02
Hoarseness	63 (49.6)	46 (51.1)	.89
Cough	123 (96.9)	88 (97.8)	1.0
Sputum production	98 (77.2)	67 (74.4)	.74
Shortness of breath	116 (91.3)	87 (96.7)	.16
Wheezing	101 (79.5)	63 (70.0)	.11
Constitutional	84 (66.1)	68 (75.6)	.18
Feverishness	61 (48.0)	58 (64.4)	.02
Rigors	23 (18.1)	26 (28.9)	.07
Confusion	18 (14.2)	4 (4.4)	.02
Temperature, °C, mean (SD)	37.3 (0.9)	37.6 (1.0)	.02
Temperature >38.0°C, No. (%)	27 (21.3)	30 (33.3)	.05
Respiratory rate, breaths/min, mean (SD)	25.3 (6.5)	23.8 (6.5)	.08
Heart rate, beats/min, mean (SD)	104 (20.7)	103.7 (21.6)	.92
Systolic blood pressure, mmHg, mean (SD)	125.8 (26.3)	123.3 (26.5)	.49
SaO_2_, %, mean (SD)	91.2 (6.3)	92.0 (4.5)	.30
Physical examination finding, No. (%)			
Wheezing	90 (70.9)	54 (60.0)	.11
Rales	36 (28.3)	24 (26.7)	.87
Rhonchi	48 (37.8)	25 (27.8)	.15
Laboratory finding, mean (SD)			
WBC, ×10^3^ cells/mL	10.3 (4.8)	9.3 (4.5)	.12
Neutrophils, %	74.0 (13.4)	73.7 (12.2)	.87
Bands, %	1.9 (6.3)	1.2 (2.9)	.33
PCT level >0.25 ng/mL, No. (%)	22 (20.0)^a^	21 (26.6)^a^	.74
Chest radiograph finding, No. (%)^b^			
No acute disease	65 (51.2)	45 (50.0)	.89
Atelectasis	29 (22.8)	14 (15.6)	.23
Infiltrate	39 (30.7)	31 (34.4)	.65
Consolidation	4 (3.2)	3 (3.3)	1.0
Edema	15 (11.8)	10 (11.1)	1.0
Effusion	9 (7.09)	7 (7.8)	1.0
Supplemental oxygen	41 (32.3)	16 (17.8)	.02
ICU admission, No. (%)	16 (12.6)	5 (5.6)	.10
Mechanical ventilation, No. (%)	7 (5.5)	1 (1.1)	.07
Antibiotic therapy, No. (%)	102 (80.3)	74 (82.2)	.86
Length of stay, d, mean (SD)	6.4 (10.3)	4.4 (3.0)	.08
In-hospital death, No. (%)	3 (2.4)	2 (2.2)	1.0

Abbreviations: HMPV, human metapneumovirus; ICU, intensive care unit; PCT, procalcitonin; RSV, respiratory syncytial virus; WBC, white blood cell.

^a^Missing values, 16 RSV, 11 HMPV.

^b^Chest radiographs could have more than 1 finding.

**Figure 1.**
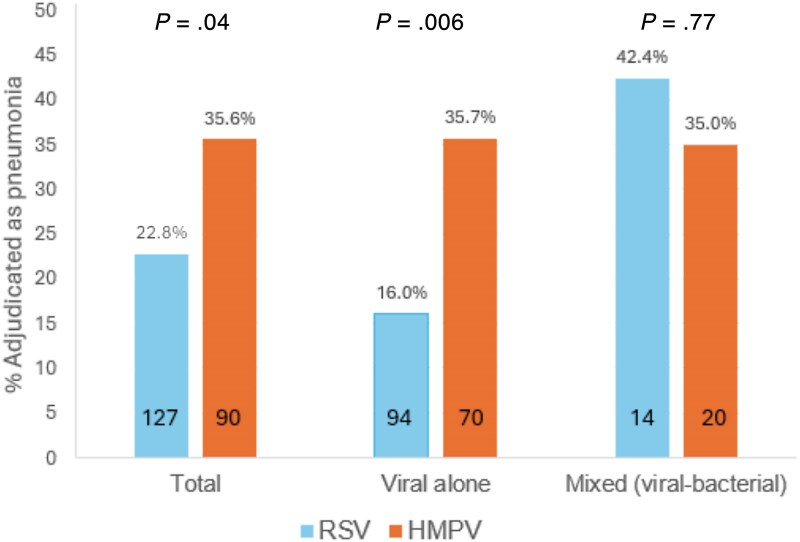
Percentage of respiratory syncytial virus (RSV) and human metapneumovirus (HMPV) cases adjudicated as pneumonia. Total group, viral alone, and mixed viral bacterial infections are displayed separately. Blue bars indicated RSV and orange represent HMPV. Numbers at the base of each bar indicates the number of subjects in each group.

Cases were adjudicated based on microbiologic data as viral infection alone or mixed viral and bacterial infection. Adjudication also consisted of review of radiographic imaging results in the context of the clinical information and determining a clinical diagnosis of pneumonia or nonpneumonic illness (see Supplementary Material). Bacterial coinfection occurred equally, with 26.0% of RSV infections and 22.2% of HMPV infections classified as mixed viral and bacterial infections (Table 4). Overall, compared with RSV, infection with HMPV was more likely to be associated with a radiographic and clinical diagnosis of pneumonia (35.6% vs 22.8%, *P* = .04) in the entire group (including bacterial coinfections). This distinction was even more pronounced in patients adjudicated as having viral infection alone (no bacterial coinfection), with 35.7% of HMPV patients judged to have pneumonia compared with only 16.0% of those with RSV (*P* = .006; Figure 1).

**Table 4.** Adjudicated Microbiologic and Pneumonia Diagnoses

Diagnosis	RSV (n = 127)	HMPV (n = 90)	*P* Value
Microbiologic diagnosis, No. (%)			
Viral infection alone	94 (74.0)	70 (77.8)	.63
Mixed viral and bacterial	33 (26.0)	20 (22.2)	
Clinical syndrome, No. (%)			
Viral infection alone			
Pneumonia	15 (16.0)	25 (35.7)	.006
Not pneumonia	79 (84.0)	45 (64.3)	
Mixed viral-bacterial infection			
Pneumonia	14 (42.4)	7 (35.0)	.77
Not pneumonia	19 (57.6)	13 (65.0)	

Abbreviations: HMPV, human metapneumovirus; RSV, respiratory syncytial virus.

### Gene Expression

**Figure 2.**
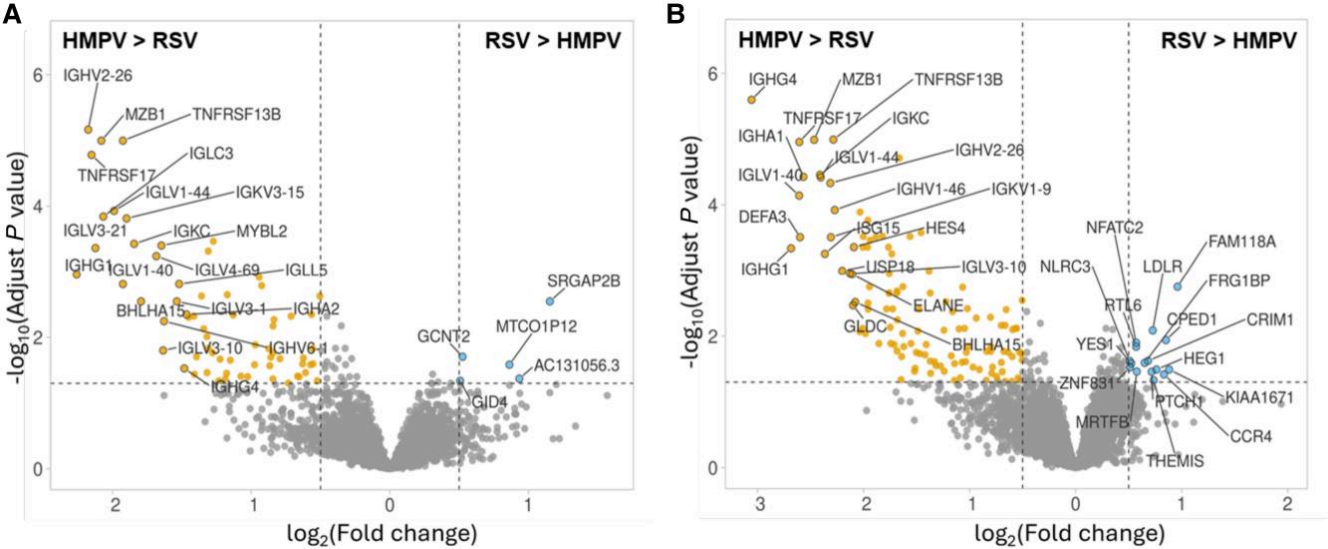
Volcano plots showing differentially expressed genes when comparing respiratory syncytial virus (RSV) versus human metapneumovirus (HMPV) in (*A*) all subjects with RSV or HMPV (including viral-bacterial coinfection), or (*B*) subjects with viral infection only (no bacterial coinfection). Highlighted genes (blue and orange circles) represent those with significant differences and greatest fold change (denoted on X-axis with adjusted *P* value on the Y-axis).

**Figure 3.**
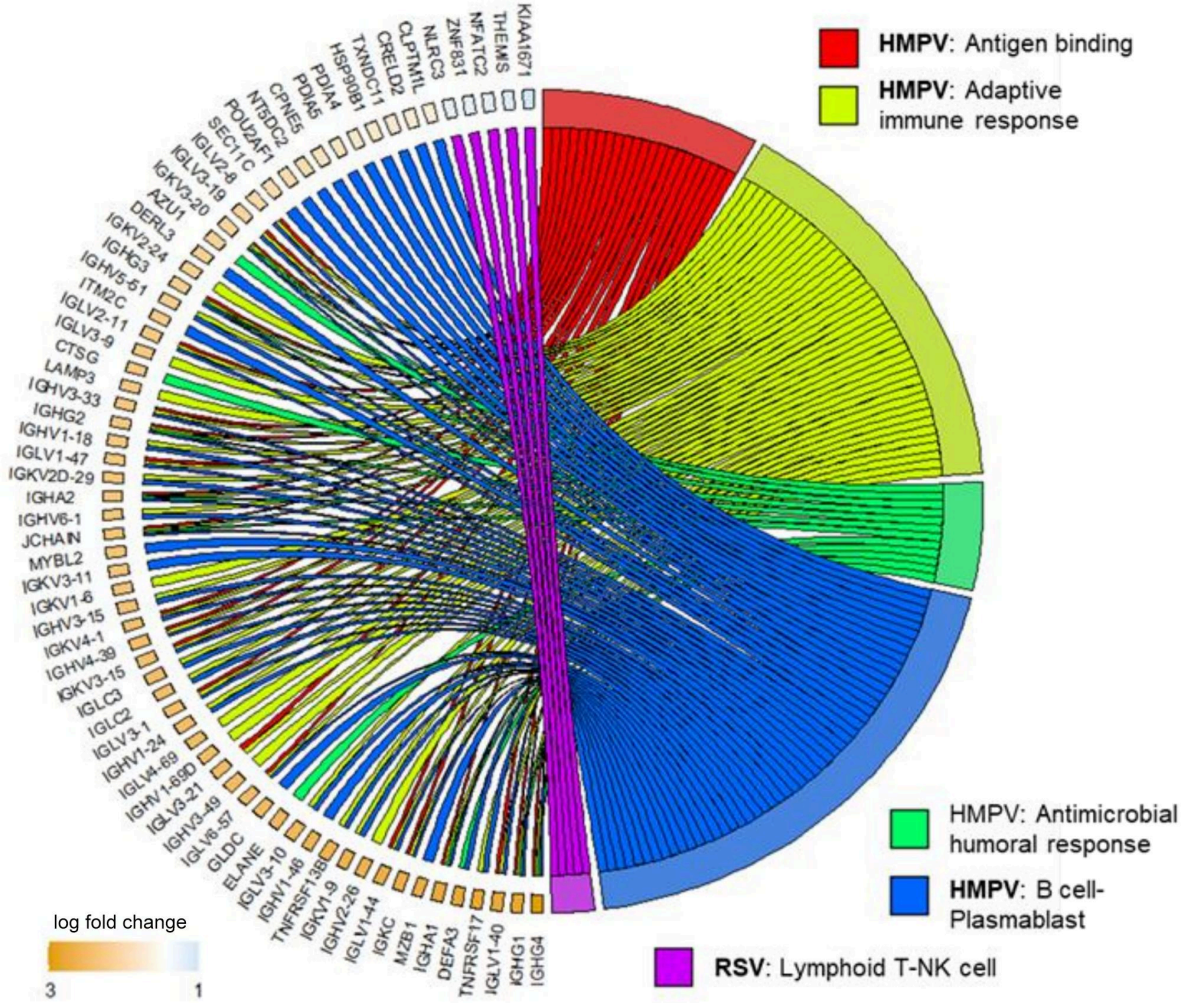
Functional characterization of gene expression changes associated with respiratory syncytial virus (RSV) versus human metapneumovirus (HMPV) infection only (excluding vial-bacterial coinfection). Selected signaling pathways significantly overrepresented by differentially expressed genes are shown on the right. The genes associated with these pathways are shown on the left, along with their color-coded relative fold change in expression.

Gene expression data were limited to subjects in study 2 with definitive microbiologic classification. This included 54 of 63 (86%) RSV and 43 of 46 (93%) HMPV cases after excluding cases for whom bacterial coinfection was uncertain. The demographic, clinical, and outcome variables for these subgroups were similar to the entire population (Supplementary Table). Importantly, the time from illness onset to sample collection in RSV and HMPV cases was identical at 6.6 (SD 4.8 days) and 6.3 (SD 5.2 days), respectively. A total of 7352 genes met quality metrics and were used for differential expression analysis. Ninety-three genes were found to have significant differences in expression (FDR < 0.05) between HMPV and RSV (Figure 2*A*). When considering only subjects without bacterial coinfection (HMPV = 35, RSV = 35) 197 differentially expressed genes (FDR < 0.05) (Figure 2*B*) were identified; 141 genes were expressed at higher levels in HMPV patients (of these 120 had log_2_ fold change > 0.5) compared to RSV-infected patients. Pathway analysis comparison of genes differentially expressed between RSV and HMPV (without bacteria coinfection) is shown in the Circos plot (Figure 3). Genes and pathways expressed at higher levels in HMPV-infected subjects included many immunoglobulin related genes involved in receptor binding to antigens, complement activation, IgM secretion, and B-cell development (ie, *IGHG4* and *IGGHV2-26*, *MZB1*, *TNFRSF17,* and *IGKC*, respectively). Genes and pathways with higher expression levels in RSV-infected subjects compared to HMPV-infected patients were associated with natural killer (NK) T-cell responses.

## DISCUSSION

Since its discovery in 2001 by Dutch scientists, HMPV has been shown to be a common cause of ARI in children with reinfection throughout adult life [1, 12, 26]. Because testing for HMPV is not commonly performed as standard of care and prospective studies are limited, the true burden of HMPV remains incompletely defined. However, most studies suggest that HMPV is a significant cause of morbidity and mortality in adults with prevalence rates in hospitalized patients 70%–100% of RSV rates [3, 26–29]. Our study was not designed as an epidemiologic study; however, using the same inclusion criteria we observed the total number of hospitalizations for HMPV were 71% of the number of RSV cases and consistent with current literature.

The primary focus of our investigation was to evaluate the similarities and differences in populations affected and the clinical presentation of illness with HMPV versus RSV, as well as differences or similarities in the host response measured by whole-blood gene expression. As closely related viruses, one might expect clinical features and gene expression to be indistinguishable. However, we found subtle differences in the clinical phenotypes and differential gene expression between the 2 viruses in hospitalized adults. Overall, the populations were similar with >97% having at least 1 chronic condition and on average >3.8 conditions per person in both groups. However, RSV patients were more frequently active smokers and tended to have more chronic cardiopulmonary conditions. In a global study of hospitalized ARI patients with HMPV and RSV, RSV patients had more chronic heart disease but otherwise the populations were very similar [30]. In a French study of hospitalized patients with influenza-like illness (ILI), the investigators found smoking was more common in RSV patients but also noted that chronic conditions were significantly more common with RSV (91% vs 81%, *P* = .04), as was the presence of chronic renal failure and cancer, compared to HMPV [28]. Lastly, our previous prospective study and a more recent large database study suggest baseline characteristic of adults hospitalized with RSV and HMPV are very similar [3, 29]. Although studies are limited, the current literature and data from our study demonstrate that the characteristics of adults at risk for severe RSV and HMPV are comparable, and thus combination vaccines targeting similar high-risk adults should be feasible.

Because signs and symptoms of HMPV and RSV have considerable overlap, syndromic diagnosis is not possible and specific viral testing is necessary [3]. However, in the current study there were some interesting differences in presenting symptoms with HMPV patients displaying more ILI symptoms, such as sore throat and fever as well as more clinical pneumonia. Higher rates of fever and pneumonia have also been described in studies of young children when comparing HMPV and RSV, although they had comparable outcomes and severity [31–35]. Observational studies of adults hospitalized with HMPV infection also note fever and pneumonia to be relatively common. However, some studies are limited by lack of comparison to RSV, retrospective study designs, and the use of an ILI case definition. For example, the study by Loubet compared clinical features of HMPV and RSV but the requirement of ILI symptoms for testing may have biased the range of symptoms observed [28].

In addition to subtle clinical differences, we found several gene expression differences in whole blood of HMPV- and RSV-infected adults. To the best of our knowledge a direct comparison of differential gene expression in HMPV- and RSV-infected adults has never been reported. Most evident was relatively greater activation of immunoglobulin and plasmablast-related genes and pathways in HMPV-infected patients. Our prior studies demonstrated that older subjects have relatively good protective neutralizing antibody responses to HMPV infection, although the same is true for older adults with RSV infection; thus, offering no clear explanation of the differential gene expressions observed [23, 24]. In previous studies of RSV and HMPV infection it has been noted that antibody responses in young adults are of lower magnitude than in older adults, and more severe RSV illness results in greater antibody responses than mild illness [36]. Consistent with the concept that young adults with mild disease may have muted antibody responses, a poor antibody response was noted in HMPV experimentally challenged young healthy adults reported by Talaat [37]. Greater antigen load or prolonged shedding with more severe illness may induce a more robust B-cell response with persistent antibody-secreting cells in circulation [21, 38]. As more HMPV-infected patients had viral pneumonia compared to RSV-infected patients, it is conceivable that this resulted in greater B-cell responses during HMPV infection. The higher rate of viral pneumonia may be due to greater intrinsic virulence or possibly new HMPV antigenic variants resulting in a mix of memory and de novo B-cell responses. Presently, it is unknown if antigen variation in HMPV infection is greater than what is observed with RSV. To explain the counterintuitive age-related antibody responses to RSV infection, it has been hypothesized that younger adults clear viral infection more efficiently with robust cellular immunity leading to a diminished antibody response [38, 39]. In our present study, genes associated with adaptive immunity were also increased in HMPV compared to RSV infection and it should be noted that cellular immunity in adults for both RSV and HMPV remains largely unexplored.

Interestingly, we did not find type I interferon gene signaling or pathway gene expression level differences between the 2 viruses. This is notable, as the most obvious differences between the viral genomes is that HMPV lacks the 2 nonstructural genes (NS1 and NS2) that encode potent inhibitors of innate interferon signaling. However, it has been demonstrated by several investigators that inhibition of innate interferon signaling is likely provided by the HMPV SH gene [40–43]. The finding of an increase in NK T-cell pathway expression in RSV-infected subjects is interesting, although it is unclear what effect this might have on proinflammatory and protective immune responses. NK T cells represent an innate T cell that can produce cytokines characteristic of both Th1 (interferon-γ, tumor necrosis factor [TNF]) and Th2 (interleukin 4 [IL-4], IL-5, IL-13) responses. In experiments using a murine model of primary HMPV and RSV infections, notable differences between the 2 viruses were noted in NK T cells, with investigators concluding that differential activation of NK T-cell function may contribute to the pathogenesis of these 2 viruses in respect to viral clearance and pathology [44].

Our study had strengths and weaknesses. The primary weakness was that both studies used to analyze HMPV and RSV were designed to develop new diagnostics for ARI and excluded immunocompromised patients, persons on antibiotics prior to admission, and required informed consent, all limiting generalizability. In addition, the populations were from studies conducted a decade apart and the latter study spanned the COVID-19 pandemic. Hospital admission patterns, diagnostics, and viral epidemiology may have been slightly different. It is also possible that gene expression in adults is affected by a lifetime of HMPV and RSV reinfections. However, because the RSV and HMPV cases were recruited with the same criteria we feel the comparison between RSV and HMPV cases is valid. In addition, the sample size was relatively small and our findings should be validated in a larger study. Finally, gene expression may vary during the course of an illness and we only collected samples for sequencing at enrollment, although duration of symptoms were similar for both viruses. Strengths of our study include the prospective nature in which patients were interviewed so that symptom data were more accurately collected than in a retrospective chart review, our population was diverse with a variety of underlying conditions, and the evaluation of host gene expression adds mechanistic insights.

In conclusion, our data are consistent with the literature that HMPV, like RSV, leads to significant illnesses in older adults. Although somewhat less frequent, HMPV infections appeared to affect a slightly healthier population and result in viral pneumonia more frequently. Notably, gene expression also differed from RSV and future studies to dissect immune responses to these viruses in adults are warranted. Development of an effective HMPV vaccine would address an unmet health need and given the overlapping epidemiology and potential vaccine target groups, a combination RSV and HMPV vaccines could be practical.

## Supplementary Material

jiaf084_Supplementary_Data

## Supplementary Data

Supplementary materials are available at *The Journal of Infectious Diseases* online (http://jid.oxfordjournals.org/). Supplementary materials consist of data provided by the author that are published to benefit the reader. The posted materials are not copyedited. The contents of all supplementary data are the sole responsibility of the authors. Questions or messages regarding errors should be addressed to the author.

## Notes

***Acknowledgments.*** The authors thank Mary Criddle, RN and Sharon Moorehead, RN for clinical assistance in enrolling subjects and collecting data.

***Financial support.*** This work was supported by the National Institutes of Health, National Institute of Allergy and Infectious Diseases (grant numbers R01AI137364 and R01AI079446); and AstraZeneca.

***Supplement sponsorship.*** This article appears as part of the supplement “Human Metapneumovirus Disease Burden in Older Adults and Implications for Adult Vaccination Strategies,” sponsored by AstraZeneca.
