# Supplementary material for: Clinical Features and Gene Expression Patterns in Adults Hospitalized With Respiratory Syncytial Virus and Human Metapneumovirus Infection
Source: J Infect Dis. 2025 Jul 16;232(Suppl 1):S37–46. doi: 10.1093/infdis/jiaf084 (PMC12265063; doi:10.1093/infdis/jiaf084)
Supplement: jiaf084_Supplementary_Data [file jiaf084_supplementary_data.zip › HMPV-RSV_Supplement-FINAL.docx]

**Supplemental Materials**

**Symptoms of Acute Respiratory Infection (ARI)**: Stuffy or runny nose, sore throat, hoarseness, new or increased cough, sputum production, wheezing or shortness of breath.

**Inclusion/Exclusion Criteria**

Study 1

| Inclusion Criteria | Exclusion Criteria |
| --- | --- |
| Acute cardiopulmonary syndrome   - Symptoms consistent with ARI - Admitting diagnosis of ARI or noninfectious acute cardiopulmonary disease | Significant Immunosuppression   - Absolute neutrophil count <500 - Known to be HIV+ with CD4 count <200 - Active chemotherapy or pulmonary radiation therapy - History of HSCT or solid organ transplant - High dose, prolonged steroids (>10mg prednisone for >2 weeks in the month preceding hospitalization) - Other immunosuppressive medications   Cavitary Lung disease  Witnessed aspiration event |
| Age ≥ 21 years | More than 24 hours of antibiotics prior to enrollment unless has positive bacterial diagnosis |
| Patient or family member can provide written consent | Inability to comply with study procedures |

Study 2

| Inclusion Criteria | Exclusion Criteria |
| --- | --- |
| Acute cardiopulmonary syndrome   - Symptoms consistent with ARI - Admitting diagnosis of ARI or noninfectious acute cardiopulmonary disease | Significant Immunosuppression   - Absolute neutrophil count <500 - Known to be HIV+ with CD4 count <200 - Active chemotherapy or pulmonary radiation therapy - History of HSCT or solid organ transplant - High dose, prolonged steroids (>10mg prednisone for >2 weeks in the month preceding hospitalization) - Other immunosuppressive medications |
| Age ≥ 18 years | More than 24 hours of antibiotics prior to enrollment unless has positive bacterial diagnosis |
| Patient or family member can provide written consent | Inability to comply with study procedures |

**Adjudication**

**Microbiologic Adjudication**

**Study 1**

Adjudication was performed by two infectious disease physicians using the criteria noted below. Unanimous agreement was required for definitive microbiologic classification.

**Virus Infection Alone**

Virus infection alone was defined as either (1) a nose and/or throat swab samples or a sputum sample positive for any virus by either RT-PCR (for all viruses), or (2) a nasal or throat swab positive by a rapid influenza antigen test, or (3) serologic criteria of a ≥4-fold rise in virus-specific IgG level (for all viruses, with exception of those detected during illness coinciding with influenza vaccination). Additional criteria involved negative results of all tests for bacteria and serum PCT values of <0.25 ng/mL on admission and day 2.

Subjects with a productive cough could be classified as viral alone only if an adequate sputum sample collected within 6 hours of initial antibiotic administration was negative for pathogens by Gram stain and culture. In addition, all febrile patients must have had negative blood cultures.

**Bacterial Infection Alone**

Bacterial infection alone was defined on the basis of negative results of viral diagnostic tests and any of the following: (1) a positive blood culture result, (2) a culture of an adequate sputum sample (<10 epithelial cells and >25 PMNs/HPF) that was positive for a respiratory pathogen, (3) a urinary antigen test positive for *S. pneumoniae* or *Legionella pneumophila,* (4) a serologic assay positive for *Streptococcus pneumoniae*, (5) a nasal or sputum sample PCR assay positive for *M. pneumoniae* or *C. pneumoniae,* or (6) a serum PCT level of ≥0.25 ng on admission or hospital day 2.

**Mixed Viral-Bacterial Infection**

A mixed viral-bacterial infection met the definitions for bacterial infection and viral infection.

For Bacterial alone or Viral +Bacterial classification, subjects who grew potential pathogens from inadequate sputum samples would not be included as bacterial positive as they may be colonized by these bacteria.

**Study 2**

Adjudication was performed by three infectious disease physicians and a pulmonary medicine specialist using the criteria noted above with the exception that serologic assays were not performed and the strict requirement that patients with productive coughs have a negative sputum culture that was obtained within 6 hours of antibiotic administration. This was done because clinical practice had de-emphasized the utility of sputum culture and because of the impact of the COVID-19 pandemic.

As above, unanimous agreement was required for definitive microbiologic classification. Only samples considered to have definitive microbiology had RNA sequencing performed.

**Clinical Pneumonia Adjudication:**

A panel of infectious disease and pulmonary specialist adjudicated all cases as to the presence of the clinical condition of pneumonia. Official radiology reports were most commonly descriptive in nature without a specific diagnosis and clinical correlation was generally suggested. Chest radiographs (CXR) and Computed Tomography (CT) reports were reviewed for any abnormalities (consolidation, other infiltrates, atelectasis, edema, pleural effusions, masses). Any case with an abnormal CXR or CT was reviewed as possible pneumonia.

Cases were adjudicated on the basis of all available information including patient interview and examination, available laboratory data and chest radiography. CXR/CT findings were compared to previous films if available and rated as unchanged or new or worsening infiltrates.

The clinical illness diagnosis was assigned based on the following definitions and consensus achieved.

Pneumonia:

Evidence of acute infection, defined as reported fever or chills, documented fever or hypothermia, leukocytosis or leukopenia, or new altered mental status with evidence of ARI, defined as new cough, or sputum production, chest pain, dyspnea, tachypnea, abnormal lung examination or respiratory failure; and new or worse infiltrate on CXR as noted above.

Non-Pneumonic ARI

AECOPD: Patients with physician diagnosis of COPD who present with sustained worsening of their condition from the stable state and beyond normal day to day variation that is acute in onset and necessitates a change in medication with the presence of URI symptoms, fever or purulent sputum. No new infiltrates on CXR.

Acute Bronchitis: Acute illness characterized primarily by cough in the presence of URI symptoms, fever or purulent sputum in the absence of underlying lung disease or new radiographic infiltrate. May occur in conjunction with an asthma exacerbation.
