# Supplementary material for: Clinical Features and Gene Expression Patterns in Adults Hospitalized With Respiratory Syncytial Virus and Human Metapneumovirus Infection
Source: J Infect Dis. 2025 Jul 16;232(Suppl 1):S37–46. doi: 10.1093/infdis/jiaf084 (PMC12265063; doi:10.1093/infdis/jiaf084)
Supplement: jiaf084_Supplementary_Data [file jiaf084_supplementary_data.zip › Supplemental_Table.docx]

**Supplemental Table. RSV and HMPV demographic and baseline characteristics in subjects included in the gene expression analysis.**

|  | RSV  (n= 54 | HMPV  (n=43) | P-Value |
| --- | --- | --- | --- |
| Age, mean (SD) | 65.4 (17.9) | 60.9 (1.68) | **.03** |
| Female sex, no. (%) | 32 (59) | 23 (54) | 0.78 |
| Illness onset to sample collection, days, mean (SD) | 6.6 (4.8) | 6.3 (5.2) | .81 |
| Race/Ethnicity, no. (%) | | | |
| White | 42 (78) | 30 (70) | NS |
| Black | 12 (22) | 12 (28) | NS |
| Non-Hispanic | 47 (87) | 38 (88) | NS |
| Active tobacco use, no. (%) | 13 (24) | 9 (21) | NS |
| Chronic medical conditions, no. (%) | | | |
| COPD | 23 (43) | 12 (21) | .14 |
| Asthma | 20 (37) | 17 (40) | NS |
| Coronary artery disease | 14 (26) | 6 (14) | .21 |
| Congestive heart failure | 14 (26) | 5 (12) | .12 |
| Diabetes mellitus | 19 (35) | 14 (32) | NS |
| Chronic kidney disease | 5 (9) | 4 (9) | NS |
| Clinical characteristics; no. (%) | | | |
| Sore throat | 16 (30) | 25 (58) | **.007** |
| Cough | 53 (98) | 43 (100) | NS |
| Wheezing | 44 (82) | 32 (74) | NS |
| Rigors | 13 (24) | 17 (40) | **.02** |
| Temperature, ^o^C; mean (SD) | 37.1 (0.9) | 37.8 (1.0) | **.0001** |
| Temperature ≥ 38^o^C | 8 (15) | 20 (47) | **.001** |
| Outcome |  |  |  |
| Pneumonia in viral alone group | 7 (20)^a^ | 16 (46)^b^ | **.04** |
| ICU care | 9 (17) | 3 (7) | .22 |
| Length of stay, days median (IQR) | 5 (2,7) | 4 (2,6) | .11 |

^a^ denominator for RSV infected, viral alone n = 35

^b^ denominator for HMPV infected, viral alone n = 35
